# Long-term oral blonanserin treatment for schizophrenia: a review of Japanese long-term studies

**DOI:** 10.1186/s12991-021-00361-3

**Published:** 2021-09-07

**Authors:** Mitsukuni Murasaki, Yoshifumi Inoue, Hiroshi Nakamura, Toshihiko Kinoshita

**Affiliations:** 1 Institute of CNS Pharmacology, Kanagawa, Japan; 2 Medical Affairs, Sumitomo Dainippon Pharma Co, Ltd, 1-13-1 Kyobashi, Chuo, Tokyo 104-8356 Japan; 3 Department of Neuropsychiatry, Kansai Medical University, Osaka, Japan

**Keywords:** Atypical antipsychotics, Blonanserin, Long-term treatment, Schizophrenia, Dopamine D3 receptor antagonist

## Abstract

In general, the course of schizophrenia is chronic accompanied not only by positive and negative symptoms but also by cognitive dysfunction associated with psychosocial disability, and thus treatment combining antipsychotics and psychological therapy is considered promising. This review focused on two prospective, open-label, multicenter, phase 3 long-term studies for approval of oral blonanserin for the treatment of schizophrenia. These two studies included both inpatients and outpatients with variable disease duration or symptom prominence according to the Positive and Negative Syndrome Scale (PANSS). The selected two studies consisted of almost the same study schedule and eligibility criteria but different protocols regarding prior medications and concomitant antipsychotics. The proportion of patients who had a baseline PANSS negative score higher than the positive score was 82.2 and 67.2% in the two studies. In both studies, patients with an illness duration of ≥ 10 years were the most common. Based on the clinical symptoms at baseline, the physician determined the treatment: blonanserin monotherapy, blonanserin in combination with the existing antipsychotic medication, or therapy simplified to haloperidol together with blonanserin. The 28-week completion rate for long-term blonanserin treatment was high in both studies (82.2 and 78.7%). The types of adverse events in both studies were similar to those in the preceding 8-week randomized, active-controlled studies in Japan, which were included in the application package for the approval of oral blonanserin for patients with schizophrenia. Long-term blonanserin use did not increase the risk of extrapyramidal symptoms but reduced the dose of antiparkinsonian drugs, minimally affecting functioning. In both studies, the PANSS total score, positive score, and negative score were improved at the last observation carried forward compared with those at baseline. In conclusion, blonanserin is useful for long-term treatment of chronic schizophrenic patients when the appropriate management of clinical symptoms and adverse drug reactions are applied. Blonanserin might represent a promising treatment option that partially or completely relieves patients with chronic schizophrenia of polypharmacy. Blonanserin may possibly fit both the current real-world clinical setting and the currently recommended approach to antipsychotic medication.

## Background

The combination of pharmacotherapy using antipsychotics and psychological therapy, such as psychosocial rehabilitation, is considered a promising treatment option for improving cognitive and social functioning as well as the psychiatric symptoms of schizophrenia [1]. Schizophrenia is a chronic, debilitating psychiatric disorder with positive and negative symptoms as well as cognitive dysfunction and significant impairment in psychosocial functioning [2–4]. Furthermore, negative symptoms represent an unmet need in treating schizophrenia, as they are strongly associated with poor social functions [5]. The functional disability related to schizophrenia is even associated worldwide with stigma and discrimination which seriously interfere with social recovery of the patients. In particular, the genetic explanation of psychosis is frequently associated with stigmatizing attitudes. In a survey of medical students, medical doctors, nurses and psychiatric outpatients, it is worrying that medical students reported the highest level of perceived stigmatizing attitudes, such as assumptions of dangerousness, unpredictability and the desire for social distance regardless of the preferred aetiological model underlying schizophrenia [6]. On the other hand, recent studies using voxel-based morphometry approaches have shown that reductions in the superior temporal gyrus and cerebellum can be interpreted as markers of a first onset of the illness [7]. While certain genetic vulnerability is involved in the pathogenesis of schizophrenia, longitudinal studies are needed to elucidate the interaction between genetic risks and environmental/social effects. Heritability, as an index of genetic influence, may have limited explanatory power unless viewed in the context of interaction with environmental/social effects [8].

To facilitate positive discussion on early medical intervention in patients with early psychosis, further evidence should be accumulated to clarify the relationship between genetic vulnerability and environmental/social effects in the pathogenesis of schizophrenia.

As schizophrenia has a chronic course and requires long-term treatment, antipsychotics that could be prescribed long term and that could enhance social functioning or daily activities without adverse side effects and without disturbing the effect of psychological therapies are desirable. The Japanese Guideline for Pharmacological Therapy of Schizophrenia strongly recommends the continuous use of antipsychotics, because their discontinuation may lead to the progression or relapse of psychiatric symptoms [9]. A systematic review of clinical trials regarding first-episode psychosis stated the importance of reducing the discontinuation rate to enable the remission of psychiatric symptoms and improvement of functional recovery [10]. Preferably, antipsychotics are selected and used for a long time so as not to induce tardive dyskinesia or treatment resistance associated with dopamine supersensitivity psychosis [11].

Blonanserin is a relatively new second-generation antipsychotic agent (SGA) for schizophrenia that has been approved in Japan, South Korea, and China. Blonanserin exhibits high receptor selectivity, being a potent full antagonist of the dopamine D_2_, D_3_, and serotonin 5-HT_2A_ receptors and exhibiting low or negligible affinity for the dopamine D_1_, serotonin 5-HT_2C_, adrenaline α1, histamine H_1_, and muscarinic M_1_ and M_3_ receptors [12]. In addition, positron emission tomography study using [^11^C]-( +)-PHNO as a tracer revealed that, at a clinical dose, blonanserin occupied dopamine D_3_ receptors to the same degree as D_2_ receptors in healthy subjects [13]. The superiority of blonanserin over placebo in relation to the primary efficacy endpoint has been demonstrated in a randomized, double-blind, placebo-controlled study in patients with schizophrenia [14]. A systematic review and meta-analysis of randomized controlled studies comparing blonanserin with other antipsychotics demonstrated that the efficacy of blonanserin is comparable to that of other SGAs and even higher regarding the negative symptoms [15]. In another meta-analysis of 10 randomized controlled studies, the overall safety outcome did not differ between blonanserin and other antipsychotics, including risperidone or aripiprazole, with some variations regarding adverse events, e.g., akathisia, extrapyramidal symptoms, prolactin levels, or weight gain [16].

The therapeutic efficacy of blonanserin was confirmed in both short-term and long-term treatments [17]; however, the concomitant use of antipsychotics is generally prohibited in short-term, randomized controlled studies. This current review focused on two prospective, open-label, long-term, phase 3 studies in Japanese patients with schizophrenia who, depending on their clinical symptoms, received blonanserin as monotherapy or combination therapy with other antipsychotics. Compared with randomized active-controlled studies, as the inclusion/exclusion criteria were less stringent, patients with more variable pathological conditions of schizophrenia were included, e.g., inpatients as well as outpatients and patients with different disease durations or symptom prominence according to PANSS, and the limitations regarding the concomitant drugs or eligibility were smaller in these two studies. Moreover, although these two studies were almost similar in the study design, they had difference in the protocols for prior medication and concomitant antipsychotic use, which reflect both conventional antipsychotic polypharmacy and currently recommended monotherapy in the real-world clinical settings. Here, we present the safety and efficacy of long-term blonanserin treatment and discuss the potential therapeutic benefit of blonanserin.

## Methods

### Overview of the studies

Two prospective, open-label, multicenter, phase 3 studies were conducted to evaluate the long-term safety and efficacy of blonanserin in patients with schizophrenia. Both studies were conducted in accordance with good clinical practice and local regulatory requirements. The BNS01 study was conducted nationwide in Japan between December 1998 and April 2002 [18]. The BNS02 study was conducted in the Kanagawa region of Japan between March 1998 and September 2000 [19]. The same inclusion/exclusion criteria were applied. Patients were eligible if they were aged 16 years or older and met the F20 schizophrenia criteria of the International Classification of Diseases 10, Diagnostic Criteria for Research. Patients were excluded if they had a history or presence of convulsive disease, organic brain disease, malignant syndrome, debilitation by dehydration or malnutrition, or high risk of self-harm or suicide attempt. Nevertheless, the protocols for prior medication and concomitant antipsychotic use differed between the BNS01 and BNS02 studies (Table 1).

**Table 1:** Concomitant drugs

Study protocol for concomitant drugs		
Concomitant use of antipsychotics		
BNS01 study	Based on the clinical symptoms, physicians determined the concomitant patterns (1) or (2)	
	Pattern (1) No prior antipsychotics, or prior antipsychotics could be switched to BNS monotherapy: BNS monotherapy was started If symptoms could not be controlled by dose adjustment of BNS, concomitant use of antipsychotics was permitted	Pattern (2) Prior antipsychotics could not be discontinued: BNS was added to the prior antipsychotics Continuous use of prior antipsychotics was permitted, but risperidone, quetiapine fumarate, perospirone hydrochloride and olanzapine were prohibited
	Following antipsychotics were prohibited: risperidone, quetiapine fumarate, perospirone hydrochloride, and olanzapine	Depending on the symptom severity, concomitant antipsychotics were reduced and discontinued if possible
	Concomitant antipsychotics were reduced/discontinued if the symptoms were relieved	If symptoms could not be controlled by dose adjustment of BNS, additional antipsychotic use (including dose increase) was permitted
BNS02 study	Basically, BNS was administered as monotherapy. Concomitant antipsychotics were prohibited except for HAL	
	Pattern (1) No prior antipsychotics, or prior antipsychotics could be switched to BNS monotherapy: BNS monotherapy was started If symptoms could not be controlled by dose adjustment of BNS, concomitant use of HAL was permitted Concomitant HAL was reduced/discontinued when the symptoms were relieved	Pattern (2) Prior antipsychotics could not be discontinued: Regimen was simplified to HAL (up to 12 mg/day) while considering chlorpromazine equivalent dosage If HAL could not be discontinued at the start of BNS, concomitant use of HAL up to 12 mg/day was permitted HAL was discontinued by week 12 while adjusting the dose of BNS If necessary, concomitant use of HAL was allowed after week 12 Concomitant HAL was reduced/discontinued when the symptoms were relieved
Concomitant use of antiparkinsonian drugs		
BNS01 study & BNS02 study	If an antiparkinsonian drug was used before the start of BNS, continued use of it was permitted; otherwise its use was prohibited. If extrapyramidal symptoms developed or worsened, antiparkinsonian drugs could be used appropriately. In BNS01 study, antiparkinsonian drugs were tapered off and terminated if possible for patients with a score of < 2 for each of the seven Drug Induced Extra-Pyramidal Symptoms Scale (DIEPSS) items	
Concomitant use of other psychotropics		
BNS01 study & BNS02 study	If a psychotropic drug was used before the start of BNS, the continuous use of it was permitted. If symptoms such as insomnia developed or worsened, the use of psychotropic drugs was permitted as appropriate. In BNS02 study, concomitant use of vegetamin or levomepromazine was prohibited	

*BNS* blonanserin, *HAL* haloperidol

### Blonanserin treatment

In both studies, eligible patients orally received 8–24 mg/day of blonanserin (Sumitomo Dainippon Pharma Co, Ltd) twice daily for 26–56 weeks. The starting dose was 8 mg/day and thereafter adjusted for each patient within the range of 8–24 mg/day (in increments of 2–8 mg/day). For elderly patients (≥ 65 years of age), precautionary safety measures were taken, i.e., reducing the initial dose to 4 mg/day.

### Concomitant drugs

#### Antipsychotics

The protocol for concomitant antipsychotic use is shown in Table 1. The physician determined the treatment patterns based on the clinical symptoms or prior medication; blonanserin was started as monotherapy or added as combination therapy to the antipsychotics already in use. In the BNS01 study, concomitant SGA use was prohibited. Depending on the symptom severity, the dose of the concomitant antipsychotic drugs was gradually reduced and terminated if possible. In the BNS02 study, the only accepted concomitant antipsychotic was haloperidol. Any other prescribed antipsychotics were switched to haloperidol alone at < 12 mg/day, in consideration of the chlorpromazine equivalent dose. Thereafter, blonanserin monotherapy was initiated. However, if physicians determined that haloperidol could not be discontinued due to uncontrolled symptoms, haloperidol was reduced/discontinued and switched to blonanserin by week 12 only if the symptoms were relieved.

### Antiparkinsonian drugs

Concomitant use of antiparkinsonian drugs was permitted according to the rules, as shown in Table 1; however, these drugs were gradually discontinued, if possible. The dose of antiparkinsonian drugs was carefully reduced, because malignant syndrome can occur in patients receiving high doses of antiparkinsonian drugs.

#### Other psychotropic drugs (hypnotics, anxiolytics, and antidepressants)

Concomitant use of psychotropic drugs was permitted according to the rules, as shown in Table 1. The dosing regimens of these drugs were not changed during the study as far as possible.

## Results

### Patient characteristics

The patient characteristics are shown in Table 2. In the BNS01 study, one patient (out of 322 patients) withdrew consent before the initiation of the study. Of the patients receiving blonanserin, 82.2% (263/321) exhibited negative symptom dominance, i.e., had a higher negative than positive score according to the Positive and Negative Syndrome Scale (PANSS). Furthermore, 72.0% of patients (231/321) had been hospitalized for a long period of time (i.e., their illness duration was > 10 years). In the BNS02 study, 61 patients were enrolled and received blonanserin. The proportion of patients with negative symptom dominance was 67.2% (41/61). In both studies, patients with an illness duration of ≥ 10 years predominated. Most of the patients were receiving antiparkinsonian drugs and antipsychotics at baseline.

**Table 2:** Patient’s characteristics at baseline

	BNS01 study		BNS02 study	
	*N* = 321 (322)^a^		*N* = 61 (61)	
	*n*	(%)	*n*	(%)
Sex				
Man	200	62.3	28	45.9
Age				
Mean [SD]	45.2 [14.0]		42.5 [15.2]	
Weight				
Mean [SD]	62.6 [13.3]		59.9 [10.8]	
Illness duration (year)				
< 3	34	(10.6)	15	(24.6)
3 ≤ < 10	54	(16.8)	16	(26.3)
10 ≤	231	(72.0)	28	(45.9)
Unknown	2	(0.6)	2	(3.3)
Inpatients	214	(66.7)	18	(29.5)
Symptomatology at baseline				
Hallucinations and delusions	62	(19.3)	18	(29.5)
Delusions	25	(7.8)	9	(14.8)
Deficiency of spontaneity and apathy I (e.g., fresh hebephrenic type)	15	(4.7)	2	(3.3)
Deficiency of spontaneity and apathy II (e.g., chronic type, fixed symptom type)	200	(62.3)	24	(39.3)
Neurosis-like state	8	(2.5)	1	(1.6)
Depressive state	6	(1.9)	5	(8.2)
Others	5	(1.6)	2	(3.3)
Predominant symptoms at baseline				
Predominant positive symptoms	45	(14.1)	15	(24.6)
No predominant	12	(3.8)	5	(8.2)
Predominant negative symptoms	263	(82.2)	41	(67.2)
Pre-medicated antipsychotics				
Yes	318	(99.1)	57	(93.8)
Pre-medicated antiparkinsonian				
Yes	297	(92.5)	56	(91.8)
PANSS total at baseline				
Mean [SD]	79.5 [24.5]		69.8 [22.2]	

^**a**^One withdrew consent before the initiation of study treatment

*SD* standard deviation

### Completed cases and reasons for discontinuation

The completion rate and reasons for discontinuation are shown in Table 3. The completion rates at week 28 were 82.2% (264/321; BNS01) and 78.7% (48/61; BNS02) and at weeks 52–56 were 48.3% (155/321; BNS01) and 62.3% (38/61; BNS02). The mean durations of the study treatments were 268.2 ± 123.2 (SD) days (BNS01) and 301.9 ± 126.5 days (BNS02). The mean final and maximum doses of blonanserin were 13.0 ± 6.7 and 14.8 ± 7.2 mg/day (BNS01), respectively, and 12.8 ± 6.1 and 14.0 ± 6.1 mg/day (BNS02), respectively. Discontinuation due to the patients’ requests was the most common reason in both studies.

**Table 3:** Completed cases and reasons of discontinuation

	BNS01 study	BNS02 study
	*N* = 321	*N* = 61
Dose of BNS		
Mean final dose [SD] (mg/day)	13.0 [6.7]	12.8 [6.1]
Mean maximum dose [SD] (mg/day)	14.8 [7.2]	14.0 [6.1]
Completed cases		
Week 28 completed, n (%)	264 (82.2)	48 (78.7)
Weeks 28–52 completed, n (%)	93 (29.0)	3 (4.9%)
Weeks 52–56 completed, n (%)	155 (48.3)	38 (62.3)
Mean treatment duration [SD] (day)	268.2 [123.2]	301.9 [126.5]
Concomitant use		
Antipsychotic drugs	286 (89.1)	17 (27.9)
Antiparkinsonian drugs	299 (93.1)	55 (90.2)

*BNS* blonanserin, *SD* standard deviation

### Treatment patterns

In the BNS01 study, 89.1% of patients (286/321) concomitantly used antipsychotics, and 93.1% (299/321) used antiparkinsonian drugs. In the BNS02 study, 27.9% (17/61) of patients concomitantly used haloperidol, and 90.2% (55/61) used antiparkinsonian drugs. Of the patients taking antiparkinsonian drugs at baseline, 21.4% (63/294; BNS01) and 39.3% (22/56; BNS02) reduced or discontinued these drugs at the last observation carried forward endpoint.

### Safety

#### Adverse events

The overall adverse event incidence rates were 96.9% (311/321; BNS01) and 96.7% (59/61; BNS02) (Table 4). Common adverse events (incidence rate ≥ 20%) in both the BNS01 and BNS02 studies were insomnia, anxiety, somnolence, nasopharyngitis, hyperprolactinemia, malaise, and dizziness. Most adverse events occurred within 8 weeks after the initiation of treatment: 82.9% (266/321; BNS01) and 90.2% (55/61; BNS02). One case of death was observed in each study: suicide unrelated to blonanserin (BNS01) and recurrent breast cancer with unknown causality (BNS02). Other serious adverse events were reported in 5.6% of patients (18/321; BNS01), including hallucination, delusion, anxiety, nervousness, depression, asthenia, malaise, akathisia, gait abnormal, somnolence, insomnia, stupor, anorexia, vomiting, accidental ingestion, coma, malignant neoplasm, hemoptysis, pathological fracture, brain contusion, and subarachnoid hemorrhage, and in 8.2% of patients (5/61; BNS02), including delusion, hallucination, nervousness, and anemia. The total numbers of adverse events were 3741 (BNS01) and 678 (BNS02), but no sedation was observed, and severe events were observed in 2.35% (85/3741; BNS01) and 4.1% (28/678; BNS02) of patients.

**Table 4:** Incidence of adverse events (≥ 5% in either of the studies)

System organ class Preferred term (adverse events)	BNS01 study		BNS02 study	
	*N* = 321		*N* = 61	
	*n*	(%)	*n*	(%)
Extrapyramidal system	115	35.8	32	52.5
Akathisia	62	19.3	21	34.4
Tremor	61	19.0	17	27.9
Bradykinesia	49	15.3	10	16.4
Dyslalia	33	10.3	4	6.6
Salivary hypersecretion	29	9.0	8	13.1
Gait abnormal	26	8.1	5	8.2
Dyskinesia	21	6.5	6	9.8
Musculoskeletal stiffness	20	6.2	4	6.6
Dystonia	12	3.7	6	9.8
Psychoneurologic system				
Insomnia	119	37.1	31	50.8
Somnolence	84	26.2	24	39.3
Headache	79	24.6	12	19.7
Anxiety	75	23.4	27	44.3
Nervousness	64	19.9	14	23.0
Depression	34	10.6	16	26.2
Delusion	8	2.5	4	6.6
General symptom				
Malaise	71	22.1	13	21.3
Dizziness	66	20.6	13	21.3
Feeling hot	41	12.8	6	9.8
Asthenia	38	11.8	10	16.4
Circulatory system				
Tachycardia	26	8.1	7	11.5
Digestive system				
Constipation	75	23.4	12	19.7
Diarrhea	63	19.6	11	18.0
Nausea	62	19.3	8	13.1
Anorexia	53	16.5	9	14.8
Thirst	50	15.6	15	24.6
Abdominal pain	41	12.8	8	13.1
Increased appetite	21	6.5	5	8.2
Toothache	21	6.5	2	3.3
Vomiting	18	5.6	2	3.3
Endocrine system				
Menstrual disorder	8	2.5	5	8.2
Others				
Nasopharyngitis	116	36.1	17	27.9
Hyperprolactinemia	95	29.6	21	34.4
Back pain	39	12.1	1	1.6
Pyrexia	38	11.8	2	3.3
Hypertriglyceridemia	36	11.2	5	8.2
Creatine phosphokinase increased	34	10.6	6	9.8
Eczema	31	9.7	1	1.6
Pharyngitis	28	8.7	4	6.6
ALT (GPT) increased	28	8.7	3	4.9
Rhinitis	27	8.4	6	9.8
Chest pain	24	7.5	4	6.6
Upper respiratory tract infection	23	7.2	5	8.2
Weight increased	23	7.2	5	8.2
Arthralgia	22	6.9	1	1.6
Gamma-glutamyltransferase increased	21	6.5	2	3.3
Hypotension	21	6.5	4	6.6
Fungal skin infection	20	6.2	3	4.9
Pain	19	5.9	0	0.0
Purpura	18	5.6	1	1.6
Cough	18	5.6	2	3.3
Hypertension	18	5.6	1	1.6
AST (GOT) increased	17	5.3	3	4.9
Conjunctivitis	16	5.0	0	0.0
Accommodation disorder	12	3.7	9	14.8
White blood cell count increased	10	3.1	8	13.1
Extrapyramidal adverse drug reactions	115	35.8	32	52.5

#### Extrapyramidal symptoms

Common extrapyramidal adverse events included akathisia (19.3%, 62 patients), tremor (19.0%, 61), bradykinesia (15.3%, 49), and dyslalia (10.3%, 33) in the BNS01 study and akathisia (34.4%, 21), tremor (27.9%, 17), and bradykinesia (16.4%, 10) in the BNS02 study. The incidence of extrapyramidal adverse drug reactions was 35.8% (115/321; BNS01) and 52.5% (32/61; BNS02) (Table 4).

#### Laboratory data

One patient in each study without laboratory data was excluded from the analysis. Abnormal changes in the laboratory data were reported in 212/320 patients (66.3%; BNS01) and 37/60 patients (61.7%; BNS02). Blood prolactin concentrations significantly decreased from baseline and were returning to normal with each visit in both studies. The mean changes from baseline at last observation carried forward were − 4.7 ± 21.36 ng/mL (BNS01) and − 7.1 ± 17.20 ng/mL (BNS02). The mean weight did not change significantly from baseline during the treatment period in any of the studies. No specific abnormal changes in the laboratory tests were observed in relation to the dose or duration of blonanserin treatment, and no clinically relevant electrocardiographic abnormalities were found in any of the studies.

### Efficacy

The PANSS total score was lower than baseline at each visit. Each PANSS subscale score (positive, negative, and general psychopathology subscale scores) decreased from baseline during the treatment period (Table 5).

**Table 5:** Change from baseline in PANSS scores (LOCF)

Category	BNS01 study			BNS02 study		
	*N* = 315			*N* = 59		
	Baseline, Mean [SD]	Change from baseline, Mean [SD]	95%CI lower, upper	Baseline, Mean [SD]	Change from baseline, Mean [SD]	95%CI lower, upper
Total	79.4 [24.6]	− 6.4 [16.0]	− 8.2, − 4.7	69.9 [22.5]	− 8.8 [19.0]	– 13.8, – 3.9
Positive	16.0 [6.7]	− 0.9 [4.8]	– 1.4, – 0.3	14.9 [5.8]	− 1.7 [6.1]	– 3.3, – 0.1
Negative	23.9 [8.1]	− 2.4 [4.5]	– 2.9, – 1.9	19.8 [7.8]	− 2.9 [3.9]	– 3.9, – 1.9
General psychopathology	39.8 [12.9]	− 3.1 [8.7]	– 4.1, – 2.2	35.2 [11.6]	− 4.3 [11.5]	– 7.3, – 1.3

*PANSS* positive and negative syndrome scale, *LOCF* last observation carried forward, *SD* standard deviation, *CI* confidence interval

In the combined subgroup of both study participants who used concomitant first-generation antipsychotics (note that the concomitant use of SGAs was prohibited), the mean change from baseline in PANSS total score (SD, 95% CI) was − 6.3 (16.4, − 8.2 to − 4.5), which was not much different from the change observed in the overall population of each study (Table 5).

## Discussion

In the both long-term studies, the 28-week completion rate for oral blonanserin treatment was about 80%. The types of adverse events were similar to those in the preceding 8-week randomized, active-controlled studies. Long-term blonanserin use did not increase the risk of extrapyramidal symptoms or weight gain but reduced the dose of antiparkinsonian drugs, minimally affecting functioning. In both studies, the PANSS total score, positive score, and negative score were decreased at the last observation carried forward compared with those at baseline. These results suggest that oral blonanserin is useful for long-term treatment of chronic patients with schizophrenia when the appropriate management of clinical symptoms and adverse drug reactions are applied.

In the BNS01 study, concomitant antipsychotic use was allowed except for SGAs, and most patients (89.1%) used concomitant antipsychotics at least one point during the study. In comparison, concomitant antipsychotics were limited in the BNS02 study—only haloperidol (up to 12 mg/day) was allowed, and blonanserin monotherapy was attempted by increasing its dose and tapering haloperidol. As a result, the concomitant use of antipsychotics was minimal.

Even though a clinical study is under a specific treatment, the results of the BNS02 study are closely similar to those of SGA monotherapy as the dose was adjusted according to the severity of the psychiatric symptoms. SGA monotherapy was recommended at the time the BNS02 study was performed; however, polypharmacy was still mainly adopted in clinical practice. In the BNS02 study, > 70% of patients received only blonanserin, and 27.9% of patients concomitantly used haloperidol. This suggests that blonanserin monotherapy is a potentially effective, safe, and tolerable long-term treatment for patients with schizophrenia.

The continuation rates at 28 weeks were high in both studies: 82.2% (BNS01) and 78.7% (BNS02). In the Clinical Antipsychotic Trials of Intervention Effectiveness (CATIE) study, a large-scale and long-term clinical study of SGAs for schizophrenia, the withdrawal rate was approximately 40–60% at 6 months and approximately 50–70% at 12 months [20]. These results suggest that the continuation rate of blonanserin treatment was not inferior to those of approved drugs. In addition, even if blonanserin was used in combination with other antipsychotic drugs, the discontinuation rate was not higher than that observed with blonanserin monotherapy. Therefore, this indicates that there is no major safety concern regarding combination therapy of blonanserin together with antipsychotic drugs. The post-hoc efficacy analysis of these two studies demonstrated that > 80% of patients continued blonanserin for 28 weeks in both studies. The remission rate in patients receiving blonanserin for 52 weeks was 30.3% (47/155; BNS01) and 50.0% (19/38; BNS02), suggesting improved efficacy after long-term blonanserin treatment [21].

The types of adverse events were similar to those in the previous 8-week randomized, controlled studies of blonanserin in Japan [22–25]. Most of the events occurred within 8 weeks, and there was no increase of adverse events or new emergence of late-onset-type events due to long-term administration. One suicide occurred during the BNS01 study, which was considered unrelated to blonanserin by the investigator. Almost all patients experienced adverse events, while only a few of them experienced events that resulted in the discontinuation of blonanserin treatment. Common adverse events found in these two studies, including insomnia, anxiety, and hyperprolactinemia, are commonly observed with other antipsychotics or in patients with schizophrenia [16] and were not late onset or refractory.

In the BNS01 study, the incidence of extrapyramidal adverse drug reactions was 35.8%, which was similar to that in Japanese 6–12-month studies of other SGAs [26–32]. In a network meta-analysis that compared oral blonanserin and other antipsychotics (e.g., aripiprazole, clozapine, clocapramine, haloperidol, olanzapine, mosapramine, paliperidone, perospirone, quetiapine, and risperidone), blonanserin exhibited a lower risk of extrapyramidal adverse drug reactions than that of haloperidol but a higher risk than those of olanzapine and quetiapine; there were no distinct differences with other antipsychotics [33]. The risk of akathisia was higher with blonanserin than olanzapine, and there were no clear differences with other antipsychotics. In the BNS02 study, the incidence of extrapyramidal adverse drug reactions was 52.5% and did not increase with treatment duration, despite the concomitant use of haloperidol. In the BNS01 study, 92.5% of patients were initially taking antiparkinsonian drugs, and 21.4% of these patients were able to discontinue or reduce these drugs, while approximately 8% required increased doses. Similarly, in the BNS02 study, 91.8% of patients were initially taking antiparkinsonian drugs, and 39.3% of these patients discontinued or reduced these drugs, while < 20% required increased doses. Although anticholinergic drugs may cause cognitive impairment and unpleasant peripheral anticholinergic side effects, they are commonly used to treat extrapyramidal symptoms [34, 35]. Since cognitive impairment obstructs the recovery from schizophrenia, anticholinergic drugs should be ideally avoided during the treatment of schizophrenia. Animal studies have suggested that dopamine D_3_ receptor antagonists suppress extrapyramidal symptoms induced by the continuous administration of haloperidol [36]. Thus, the reductive mechanism of antiparkinsonian drugs concomitantly used with blonanserin might relate to its inhibition of the D_3_ receptor.

It has been reported that treating schizophrenia with SGAs represents a risk for weight gain, metabolic syndrome, and cardiovascular events [37]. Weight gain is also known to be a possible consequence of discontinuing antipsychotic medication. The incidence rates of adverse weight gain observed with long-term blonanserin treatment were 7.2% (BNS01) and 8.2% (BNS02). A low incidence rate of weight gain (5%) was also observed in a 52-week study involving 200 Japanese patients received blonanserin transdermal patches [38]. A network meta-analysis demonstrated that blonanserin had the lowest risk for weight gain [33]. The low incidence of weight gain might be attributed to the receptor selectivity of blonanserin: a potent affinity for the dopamine D_2_ and D_3_ and serotonin 5-HT_2A_ receptors and low or negligible affinity for the 5-HT_2C_, 5-HT_1A_, and H_1_ receptors, which are involved in SGA-induced weight gain [39].

In both studies, the PANSS total score, positive score, negative score, and general psychopathology score decreased from baseline during the treatment periods, which indicates that the efficacy of blonanserin regarding psychiatric symptoms of schizophrenia was sustained during long-term treatment. Considering that 82.2% (BNS01) and 67.2% of patients (BNS02) exhibited negative symptom dominance, the effect of blonanserin is interesting. The limitations of this review include the following: the two studies reviewed had no comparators because of the open-label design; although the setting of the BNS01 study was close to polypharmacy, the combinations with risperidone, quetiapine, perospirone and olanzapine have not been studied yet.

It is reported that blonanserin improves verbal fluency and executive function (cognitive function), as assessed by the Brief Assessment of Cognition in Schizophrenia, Japanese-language version, as well as daily living and work skills (social function) as measured using the Life Assessment Scale for the Mentally Ill in patients with acute-phase schizophrenia [40]. In addition, blonanserin add-on therapy for refractory schizophrenic patients reduces the total dose of antipsychotic drugs and improves psychiatric symptoms, enhancing both function, as measured by the Social and Occupational Functioning Assessment Scale, and subjective well-being, as measured by the Subjective Well-being under Neuroleptic Scale [41]. These results do not follow/evaluate genetic vulnerability or changes in neuroanatomical markers, but at least suggest that treatment with blonanserin improves schizophrenia-related functional disability, and we hope that comprehensive treatment of schizophrenia, using blonanserin, will be useful in terms of improving stigma and discrimination.

In addition, in a multicenter, randomized, rater-blinded study on adjunct antipsychotic treatment for schizophrenic and dopamine-supersensitive psychotic patients, blonanserin improved psychiatric symptoms and reduced the amount of prior antipsychotics; the blonanserin monotherapy rate at the endpoint was 26.3% [18], similar to another retrospective study [17]. In the above-mentioned studies, many cases used blonanserin in combination with prior SGAs.

Recently, a new formulation of blonanserin, the transdermal patch, has been approved in Japan, which has the advantages as follows: easy supervision, no first-pass metabolism that can affect plasma levels of oral blonanserin, lower incidence of overall or extrapyramidal adverse events compared with oral blonanserin possibly due to more stable plasma blonanserin levels over long periods [42], and sustained positive patients’ attitudes (assessed by drug attitude inventory 10-item version and patient-questionnaire) to the medication with transdermal patch during long-term treatment [38].

Furthermore, blonanserin was approved in Japan for the treatment of schizophrenia in adolescents, making it the only atypical antipsychotic in Japan for the indications for adolescents with schizophrenia. It is expected that blonanserin can contribute to early medical access for patients with early psychosis.

Taken together, blonanserin, both used as monotherapy or in combination with other antipsychotics, exhibits long-term efficacy against positive and negative symptoms in patient populations who have mostly chronic and predominantly negative symptoms. Such therapy does not exhibit safety issues that would cause poor treatment adherence.

## Conclusions

Sustainable efficacy of blonanserin is useful for long-term continuous use in chronic patients. It is considered to be a promising treatment option for eliminating or reducing polypharmacy while managing adverse drug reactions appropriately. Given the high continuation rate in various settings, including monotherapy or combination therapy with other antipsychotic drugs, blonanserin may possibly fit both the current real-world clinical setting and the currently recommended approach to antipsychotic medication. In addition, because blonanserin is the only atypical antipsychotic for adolescent schizophrenia approved in Japan, it is expected that blonanserin can contribute to early access to medical intervention for patients with early psychosis. Furthermore, a new formulation of blonanserin, the transdermal patch, was approved in Japan for the treatment of schizophrenia [42]. This new option is further expected to contribute to the treatment adherence in the continuous treatment of patients with schizophrenia.

## Data Availability

Data sharing is not applicable to this article as no data sets were generated or analyzed during the current study. In addition, information about public data sharing was not included in the informed consent form of the two studies included in this review.

## Abbreviations

PANSS: Positive and Negative Syndrome Scale
SGA: Second-generation antipsychotic agent
